# Atypical symptoms and delayed diagnosis are more common in elderly patients with benign paroxysmal positional vertigo: a single-center study

**DOI:** 10.3389/fneur.2025.1575816

**Published:** 2025-04-24

**Authors:** Hongyan Li, Yanping Tong, Yan Wang, Meimei Chen, Yu Cui, Xiaolin Wu, Yi Ju

**Affiliations:** ^1^Department of Otorhinolaryngology Head and Neck Surgery, Beijing Tiantan Hospital, Capital Medical University, Beijing, China; ^2^Clinical Center for Vertigo and Balance Disturbance, Capital Medical University, Beijing, China; ^3^Department of Traditional Chinese Medicine, Beijing Tiantan Hospital, Capital Medical University, Beijing, China; ^4^Department of Neurology, Beijing Tiantan Hospital, China National Clinical Research Center for Neurological Diseases, Capital Medical University, Beijing, China

**Keywords:** benign paroxysmal positional vertigo, dizziness, vestibular symptoms, diagnosis, elderly

## Abstract

**Objective:**

To analyze the clinical characteristics of elderly patients with benign paroxysmal positional vertigo (BPPV), to provide a basis for the accurate diagnosis of elderly BPPV patients.

**Methods:**

A retrospective case-control study was conducted to evaluate the clinical data of 12,282 patients diagnosed with BPPV who received treatment at the Vertigo Clinical Diagnosis and Treatment Research Center of Beijing Tiantan Hospital, affiliated with Capital Medical University, between January 2020 and June 2024. Patients were categorized into an elderly group (≥65 years old) and a young-to-middle-aged group (<65 years old). Risk factors, clinical manifestations, and the interval from symptom onset to diagnosis were systematically compared and analyzed.

**Results:**

The mean age of elderly BPPV patients was 71.2 ± 5.4 years, with a male-to-female ratio of approximately 1:2. The posterior semicircular canal was the most commonly affected site (62.3%) in elderly patients, and the incidence of horizontal semicircular canal canalolithiasis was higher in the elderly group compared to the young-to-middle-aged group (16.2% vs. 13.6%, *p* < 0.001). The prevalence of arteriosclerosis-related risk factors (including hypertension, diabetes, hyperlipidemia, and coronary heart disease) was significantly higher (*p* < 0.001). Elderly patients with BPPV were more likely to experience atypical symptom (40.5% vs. 35.5%, *p* < 0.001), isolated vestibular symptom episodes lasting more than 1 min (23.1% vs. 21.4%, *p* = 0.039), and symptoms accompanied by tinnitus (21.0% vs. 18.8%, *p* = 0.004) and hearing loss (12.7% vs. 8.6%, *p* < 0.001). The proportion of elderly patients whose vertigo was triggered by typical head or body position changes was significantly lower than that in the younger group (35.3% vs. 39.7%, *p* < 0.001). Additionally, the time from symptom onset to diagnosis was positively correlated with age (r = 0.122, *p* < 0.001), and a significantly higher proportion of elderly patients experienced a diagnostic delay exceeding 14 days (35.4% vs. 28.4%, *p* < 0.001).

**Conclusion:**

Elderly patients with BPPV are more likely to present with atypical symptoms, fewer episodes of position-induced vertigo, and a longer symptom duration. Delayed diagnosis is more prevalent among elderly patients.

## 1 Introduction

Benign paroxysmal positional vertigo (BPPV) is a common cause of vertigo, especially among elderly patients (1), significantly affecting their daily activities and increasing the risk of falls and fractures (2). The lifetime prevalence of BPPV is approximately 2.4%, rising to 3.4% in individuals aged 60 and above, and reaching 4.5% in those over 75 (3, 4). The diagnosis of BPPV primarily depends on characteristic clinical symptoms and the presence of distinctive nystagmus observed during positional testing (5, 6). However, studies indicate that elderly patients frequently exhibit atypical symptoms (7), which can lead to delayed diagnosis or even misdiagnosis. Despite the clinical significance of this issue, large-scale studies analyzing the clinical characteristics of elderly BPPV patients remain limited. This study aims to compare the clinical characteristics of elderly and young-to-middle-aged BPPV patients through structured vestibular symptoms history interviews, providing insights for the early identification of atypical symptoms in elderly individuals with BPPV.

## 2 Methods

This retrospective case–control study analyzed data from the Vertigo and Balance Data System (VBDS) database at the Dizziness Clinical Diagnosis and Treatment Research Center, affiliated with Beijing Tiantan Hospital, Capital Medical University, between January 2020 and June 2024. BPPV was diagnosed using the SRM-IV BPPV diagnostic and treatment system.

### 2.1 Inclusion criteria

(1) Definitive diagnosis of BPPV based on the 2015 Bárány Society diagnostic criteria (5); (2) Age ≥18 years; (3) Completion of a structured vestibular symptoms history questionnaire with comprehensive clinical data.

### 2.2 Exclusion criteria

(1) Acute phase of cerebrovascular disease; (2) Patients with severe central nervous system disorders, psychiatric illnesses, or serious internal medical conditions that prevent effective participation in medical history collection, structured questionnaire surveys, and positional tests; (3) Patients with multiple recurrent visits; (4) Patients in the acute or symptomatic phase of other vestibular disorders.

A total of 12,282 confirmed cases were included. Clinical data were extracted from the BPPV sub-database of the VBDS database, including gender, age, time from the initial onset of vestibular symptoms to diagnosis, vestibular symptoms characteristics, accompanying symptoms (e.g., headache, autonomic symptoms, auditory symptoms, sleep disorders, neck/shoulder pain, etc.), vestibular symptoms triggers, and medical history.

According to the World Health Organization (WHO) age classification standards, patients were divided into two groups: those aged ≥ 65 years were classified as the elderly group, while those < 65 years were categorized as the young-to-middle-aged group. Based on different vestibular symptoms, patients were further classified into the vertigo group or the atypical symptoms group. Definitions of vertigo and atypical symptoms were adopted from the Bárány Society’s ‘Classification of Vestibular Symptoms’ (8). Vertigo was defined as external rotatory vertigo, whereas atypical symptoms encompassed non-rotational vertigo, dizziness, vestibular visual symptoms, and postural symptoms.

### 2.3 Quality control

1. Data collection: Patients’ clinical information and vestibular function test results were recorded by physicians trained in the VBDS database’s structured questionnaire system.
2. BPPV Diagnosis and Repositioning Treatment Procedures: Experienced physicians, proficient in the fully automated SRM-IV BPPV diagnostic and treatment system, performed both the diagnosis and repositioning procedures, ensuring accuracy and consistency in diagnostic and therapeutic outcomes.

### 2.4 Statistical analysis

Statistical analyses were performed using SPSS software version 27.0. Categorical data were presented as counts and percentages [*n* (%)], and comparisons were conducted using the χ^2^ test or Fisher’s exact test, as appropriate. Continuous variables were expressed as mean ± standard deviation (Mean ±SD). For normally distributed data, a t-test was used to compare two groups, whereas non-parametric tests were applied for non-normally distributed data. Spearman’s correlation coefficient test was used to evaluate associations. All statistical tests were two-tailed, with *p* < 0.05 considered statistically significant.

## 3 Results

### 3.1 Comparison of baseline information between elderly and young-to-middle-aged BPPV patients

Both groups had a predominance of female patients, with a male-to-female ratio of approximately 1:2; however, the difference between groups was not statistically significant. In terms of medical history, the elderly group had a higher proportion of patients with hypertension, diabetes, hyperlipidemia, and coronary heart disease compared to the young-to-middle-aged group. Conversely, a history of Menière disease, motion sickness, and headaches was less common in the elderly group than in the young-to-middle-aged group, with statistically significant differences (*p* < 0.001). Regarding the affected semicircular canals, posterior canal BPPV (canalolithiasis and cupulolithiasis) was the most frequently observed type in both groups (62.3% vs. 66.3%), followed by horizontal canal (canalolithiasis and cupulolithiasis) BPPV (22.3% vs.19.2%). However, the incidence of horizontal canal canalolithiasis was significantly higher in the elderly group (16.2% vs. 13.6%, *p* < 0.001) (Table 1).

**Table 1:** Baseline information of elderly and young-to-middle-aged BPPV patients.

Variables	Total population	Age < 65 years	Age ≥ 65 years	*p*-value
	(*n* = 12,282)	(*n* = 8,731)	(*n* = 3,551)	
Age (Mean ± SD, years)	55.6 ± 14.1	49.3 ± 11.3	71.2 ± 5.4	
Gender [*n* (%)]				
Female	8,242 (67.1)	5,890 (67.5)	2,352 (66.2)	0.190
Medical history [*n* (%)]				
Hypertension	3,923 (31.9)	2,439 (27.9)	1,484 (41.8)	<0.001
Diabetes	1,027 (8.4)	559 (6.4)	468 (13.2)	<0.001
Hyperlipidemia	962 (7.8)	596 (6.8)	366 (10.3)	<0.001
Coronary Heart Disease	496 (4.0)	255 (2.9)	241 (6.8)	<0.001
Menière Disease	178 (1.4)	153 (1.8)	25 (0.7)	<0.001
Motion Sickness	3,881 (31.6)	2,807 (32.1)	1,074 (30.2)	0.040
BPPV	863 (7.0)	592 (6.8)	271 (7.6)	0.094
Headache	1,216 (9.9)	909 (10.4)	307 (8.6)	0.003
Affected semicircular canals [*n* (%)]				
Posterior Canal BPPV Canalolithiasis	7,394 (60.2)	5,322 (61.0)	2,072 (58.4)	0.007
Posterior Canal BPPV Cupulolithiasis	605 (4.9)	465 (5.3)	140 (3.9)	<0.001
Horizontal Canal BPPV Canalolithiasis	1,769 (14.4)	1,192 (13.6)	577 (16.2)	<0.001
Horizontal Canal BPPV Cupulolithiasis	706 (5.8)	489 (5.6)	217 (6.1)	0.271
Other BPPV^*^	1,808 (14.7)	1,263 (14.5)	545 (15.4)	0.211

^*^Other BPPV, BPPV other than posterior canal BPPV and horizontal canal BPPV, including anterior canal BPPV, unilateral or bilateral multiple canal BPPV, and position test weakly positive and unable to lateralize.

### 3.2 Comparison of clinical characteristics between elderly and young-to-middle-aged BPPV patients

Regarding vestibular symptoms in BPPV, vertigo remained the most frequently reported symptom among all patients (vertigo vs. atypical symptoms 63.0% vs. 37.0%). However, in the elderly group, the proportion of patients experiencing atypical symptoms was significantly higher than in the young-to-middle-aged group (40.5% vs. 35.5%, *p* < 0.001). Isolated episodes of vestibular symptoms lasting more than 1 min, accompanied by tinnitus and hearing loss, were more commonly observed in the elderly group. In contrast, the proportion of patients whose symptoms were triggered by typical head or body positional changes, as well as those presenting with characteristic clinical features, was significantly lower in the elderly group compared to the young-to-middle-aged group, with statistically significant differences (*p* < 0.001) (Table 2).

**Table 2:** Comparison of clinical characteristics between elderly and young-to-middle-aged BPPV patients.

Variables	Total population (*n* = 12,282)	Age < 65 years (*n* = 8,731)	Age ≥ 65 years (*n* = 3,551)	*P-*value
Vestibular symptoms				
Vertigo	7,743 (63.0)	5,630 (64.5)	2,113 (59.5)	<0.001
Atypical Symptoms	4,539 (37.0)	3,101 (35.5)	1,438 (40.5)	
Single episode of vestibular symptoms duration				
≤1 min	9,591 (78.1)	6,861 (78.6)	2,730 (76.9)	0.039
>1 min	2,691 (21.9)	1,870 (21.4)	821 (23.1)	
Typical head position or body position change induction	7,175 (58.4)	5,152 (59.0)	2,023 (57.0)	0.038
Typical clinical symptoms (vertigo + head position or body position change induction)	4,722 (38.4)	3,469 (39.7)	1,253 (35.3)	<0.001
Accompanying Symptoms [*n* (%)]				
Tinnitus	2,388 (19.4)	1,641 (18.8)	747 (21.0)	0.004
Hearing loss	1,200 (9.8)	748 (8.6)	452 (12.7)	<0.001
Neck and shoulder pain	1,391 (11.3)	1,003 (11.5)	388 (10.9)	0.374
Autonomic symptoms (nausea, vomiting, etc.)	2,409 (19.6)	1,697(19.4)	712 (20.1)	0.437
Sleep disorders	4,584 (37.3)	3,265 (37.4)	1,319 (37.1)	0.794

### 3.3 Correlation analysis between age and time from first vestibular symptoms to BPPV diagnosis

**Figure 1:**
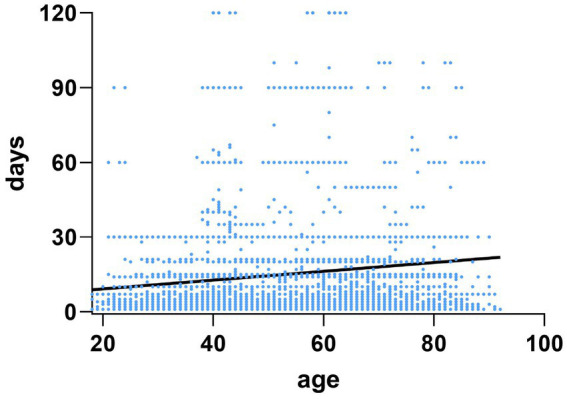
Correlation test between age and diagnosis time.

**Figure 2:**
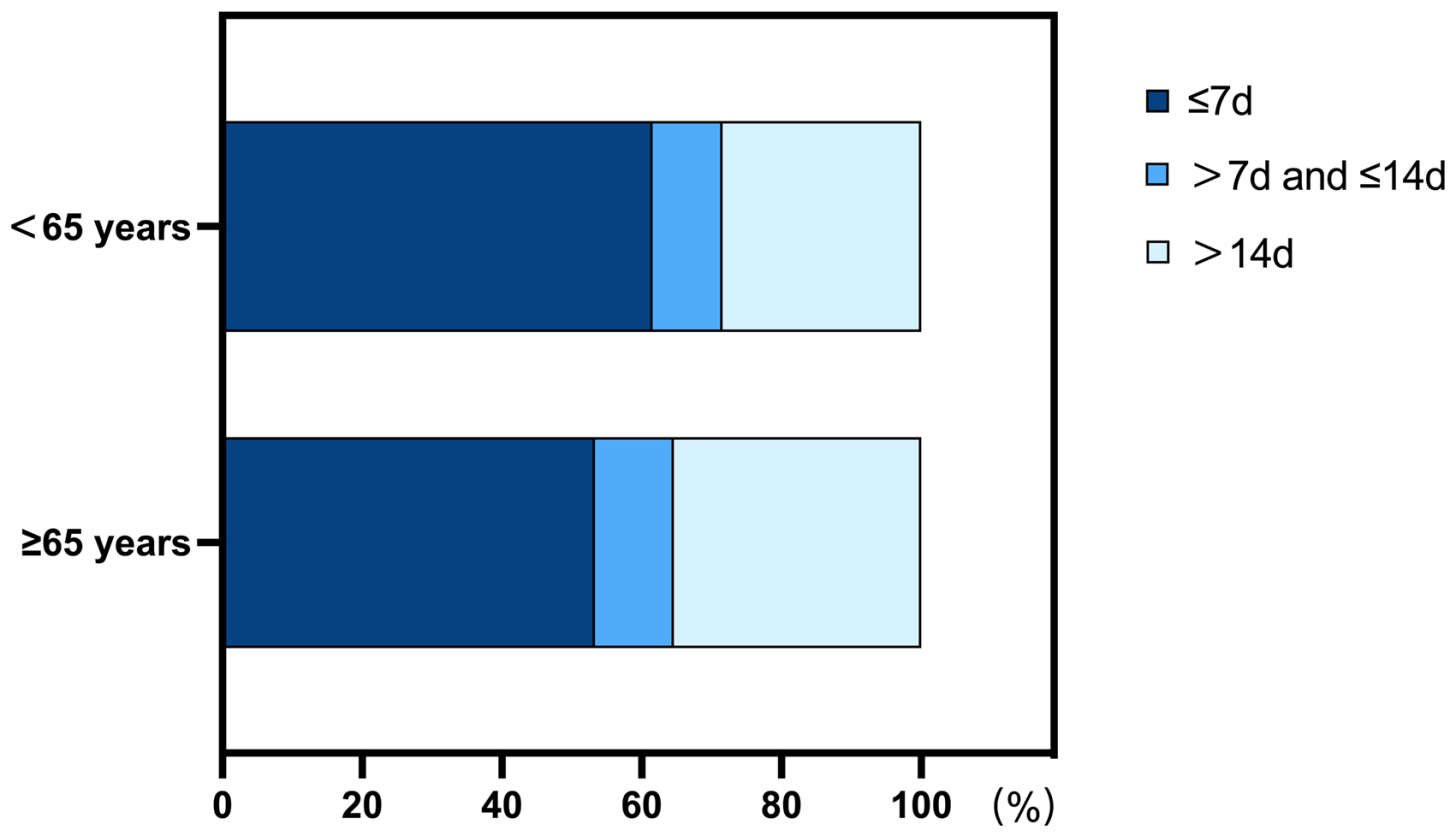
Comparison of time from first vestibular symptoms to BPPV diagnosis between elderly and young-to-middle-aged patients.

A linear positive correlation was observed between age and diagnosis time (r = 0.122, *p* < 0.001, Figure 1). Diagnosis time was categorized into three groups: ≤7 days, 7–14 days, and >14 days. The proportion of elderly patients diagnosed within≤7 days was significantly lower than that of young-to-middle-aged patients (1,891/3,551, 53.3% vs. 5,379/8,731, 61.6%; *p* < 0.001), while the proportion of patients diagnosed > 14 days was significantly higher in the elderly group compared to the young-to-middle-aged group (1,258/3,551,35.4% vs. 2,477/8,731,28.4%; *p* < 0.001) (Figure 2).

## 4 Discussion

This study demonstrates that posterior canal involvement is common in both age groups. The proportion of posterior canal was significantly lower (62.3% vs. 66.3%) and horizontal canal BPPV was more prevalent (22.3% vs. 19.2%) in elderly patients, which aligns with previous reports in the literature (9). These findings suggest that increased attention should be given to the higher incidence of horizontal semicircular canal BPPV in elderly patients. This study also found that, compared to middle-aged and young patients, elderly patients with BPPV had higher rates of comorbid hypertension, hyperlipidemia, coronary heart disease, and diabetes. They also exhibited a higher prevalence of unilateral and bilateral multi-canal BPPV. It is speculated that this may be related to the fact that elderly patients are more prone to atherosclerosis-related diseases, inner ear microcirculation disorders, inner ear degeneration, and increased otolith detachment. Additionally, these comorbid conditions may contribute to more atypical and complex clinical symptoms, leading to more complicated and delayed diagnoses (10, 11). This study also found that elderly BPPV patients had a lower proportion of headache history compared to young-to-middle-aged patients, with significant differences (*p* < 0.001). Previous research has suggested a correlation between BPPV and migraines (12). However, our database did not distinguish between different headache types, making it unclear whether these patients had a history of migraines. Given the declining incidence of vestibular headaches with age (13), further detailed analysis is required to determine if our findings reflect this trend. Additionally, the proportion of Menière disease and motion sickness history was lower in the elderly group, possibly due to age-related vestibular function decline and the peak incidence of Menière disease occurring between40 and 60 years of age (14).

Elderly BPPV patients were more likely to experience atypical symptoms, lower proportions of typical position-induced vertigo, and a longer duration of vestibular symptoms (>1 min), with significant differences (*p* < 0.001). Previous studies also reported higher incidences of non-rotational dizziness, instability, and balance disorders in elderly BPPV patients (15–17), particularly among those with multiple comorbidities (18). Several factors may contribute to these findings, including age-related structural and functional deterioration of the vestibular peripheral and central systems, neuronal loss, declines and delays in afferent pathways and central information integration (19, 20), reduced perception of vestibular symptoms (particularly vertigo), and an overall decrease in sensitivity. Additionally, elderly patients may have greater difficulty in understanding and identifying different vestibular symptoms compared to middle-aged and young individuals. These factors may contribute to the increased occurrence of atypical BPPV symptoms in elderly patients. Currently, the diagnosis of BPPV primarily relies on typical symptoms and positive positional tests (5). However, due to the atypical symptoms presentation in elderly patients, relying solely on characteristic medical history reduces the likelihood of considering BPPV in the initial diagnosis, leading to misdiagnosis, missed diagnosis, and delayed diagnosis.

In addition, our study found that tinnitus and hearing loss were more common in elderly BPPV patients, which is consistent with previous studies indicating that the prevalence of hearing loss in elderly BPPV patients is approximately 2.5 times higher than in young-to-middle-aged patients (21). Furthermore, otolith loss is more likely to occur in ears affected by tinnitus and impaired hearing (22, 23), and is believed to be associated with age-related structural and functional degeneration of the peripheral and central auditory systems, particularly within the inner ear. At the same time, the presence of these aural symptoms may also obscure or generalize the description of vestibular symptoms in elderly patients, potentially leading clinicians to shift their diagnostic focus toward other inner ear diseases that involve both vestibular and cochlear symptoms, such as Menière disease.

Previous studies have shown that diagnosis of BPPV in elderly patients is more likely to be missed or misdiagnosed, leading to delays in accurate diagnosis and treatment (18, 24, 25). This study yielded similar findings, demonstrating a positive linear correlation between age and time to diagnosis, with older patients being more prone to delayed diagnosis. The proportion of elderly patients with a diagnosis time exceeding14 days is significantly higher than that of the young-to-middle-aged group, possibly due to the atypical symptom presentation and longer disease course in elderly BPPV patients, making their vestibular symptoms less distinctive. Additionally, age-related cognitive decline may impair the brain’s ability to recognize and process various vestibular symptoms, contributing to delayed diagnosis.

The large sample size of this study supports the finding that elderly BPPV patients exhibit more atypical symptoms, present with complex accompanying symptoms, and a higher likelihood of delayed diagnosis compared to young-to-middle-aged patients. However, this study has certain limitations. Since the data were obtained from a single center, the possibility of selection bias cannot be ruled out. Additionally, as the data were collected through a structured questionnaire, patients’ comprehension or recall bias may have impacted data accuracy. Furthermore, the analysis of accompanying symptoms could be further refined and expanded to improve specificity and depth.

## 5 Conclusion

In conclusion, since the clinical presentation of elderly BPPV patients is often more complex and atypical, positional testing should be considered for elderly patients experiencing vestibular symptoms lasting more than 14 days, even in the absence of typical BPPV manifestations. This is particularly important for patients with atypical symptoms that is not triggered by head or body positional changes, especially those with multiple risk factors for arteriosclerosis and coexisting auditory symptoms, such as tinnitus or hearing loss. Conducting positional testing in these cases can help reduce both misdiagnosis and missed diagnoses, ultimately enhancing diagnostic accuracy and reducing the negative consequences of delayed medical evaluation.

## Data Availability

The original contributions presented in the study are included in the article/supplementary material, further inquiries can be directed to the corresponding author.
